# Non-equilibrium 8*π* Josephson effect in atomic Kitaev wires

**DOI:** 10.1038/ncomms12280

**Published:** 2016-08-02

**Authors:** C. Laflamme, J. C. Budich, P. Zoller, M. Dalmonte

**Affiliations:** 1 Institute for Quantum Optics and Quantum Information of the Austrian Academy of Sciences, 6020 Innsbruck, Austria; 2 Institute for Theoretical Physics, University of Innsbruck, 6020 Innsbruck, Austria

## Abstract

The identification of fractionalized excitations, such as Majorana quasi-particles, would be a striking signal of the realization of exotic quantum states of matter. While the paramount demonstration of such excitations would be a probe of their non-Abelian statistics via controlled braiding operations, alternative proposals exist that may be easier to access experimentally. Here we identify a signature of Majorana quasi-particles, qualitatively different from the behaviour of a conventional superconductor, which can be detected in cold atom systems using alkaline-earth-like atoms. The system studied is a Kitaev wire interrupted by an extra site, which gives rise to super-exchange coupling between two Majorana-bound states. We show that this system hosts a tunable, non-equilibrium Josephson effect with a characteristic 8*π* periodicity of the Josephson current. The visibility of the 8*π* periodicity of the Josephson current is then studied including the effects of dephasing and particle losses.

## Abstract (web summary)

Detecting Majorana quasi-particles requires unambiguous and experimentally accessible fingerprints. Here, the authors demonstrate the existence of a 8*π*-periodic fractional Josephson effect in a Kitaev wire as a signature for Majorana quasi-particles, and propose a cold atom experiment for its detection.

## Graphical abstract

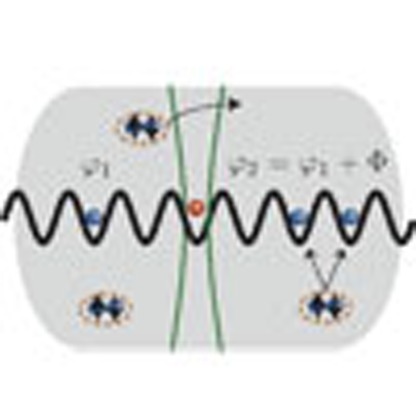

The search for observable signatures that identify exotic states of quantum matter and their fractionalized excitations has become a main focus of research in quantum physics. A paradigmatic example is the hunt for Majorana quasi-particles (MQPs) that exist at the ends of topological superconductors^1^. First experimental evidence^2^,^3^,^4^,^5^,^6^,^7^ consistent with the presence of MQPs has recently been reported in various superconducting hybrid systems^8^,^9^,^10^. While the ultimate goal is to probe the existence of non-Abelian anyons such as MQPs by performing controlled braiding operations, several possible fingerprints have been proposed that may be easier to access experimentally.

A prominent example hallmarking MQPs is the fractionalization of the Josephson effect, which can exhibit a 4*π* (half frequency) period due to a non-equilibrium population of excited states that is protected by fermion parity conservation^1^,^4^. However, a similar, though non-protected, fractionalization is also known to occur in conventional S-wave superconductors, due to the presence of accidental mid-gap states^11^,^12^. As a new signature for MQPs, here we show how a dissipationless, non-equilibrium 8*π* periodic Josephson effect occurs when two MQPs are subject to a super-exchange coupling via a controllable energy level interrupting a Kitaev chain, an effect that is not found in S-wave superconductors. In addition, we show how our model can be realized in systems of cold atoms in optical lattices, where isolation from the environment creates an ideal platform for the study of such non-equilibrium phenomena.

Our proposal is motivated by remarkable recent experimental progress with cold atom systems, including the observation of the non-equilibrium Josephson effect^13^, initially demonstrated with Bose–Einstein condensates^14^,^15^, and later observed over the Bose-Einstein Condensate (BEC)–Bardeen-Cooper-Schrieffer (BCS) crossover^16^,^17^. These results demonstrate not only the ability to measure non-equilibrium signals, but in addition, this realization of the 2*π* Josephson effect^17^ will provide a crucial piece of our implementation. More concretely, in our proposal, the starting point is an atomic realization of the Kitaev wire^18^,^19^,^20^,^21^, here using a system of alkaline earth atoms (AEAs) coupled to a BEC reservoir (Fig. 1b). AEAs allow the creation of a controllable extra site by means of species-dependent potentials^22^, while the reservoir allows both the implementation of the Kitaev wire and the modification of the Josephson phase via an underlying Josephson effect of the reservoir itself. In addition, we investigate the visibility of this effect by studying the transient dynamics of the Josephson current in the presence of imperfections, including various dissipation mechanisms (single-particle losses and dephasing) captured by a quantum master equation. Our simulations support not only the observability of the 8*π* effect, but further underline how this signature is characteristic of MQPs: while 4*π* peaks in the Fourier signal cannot be distinguished from those arising from mid-gap states in an ordinary S-wave SC, and peaks at 4*π*, 2*π* and zero frequency can be enhanced from dissipation, the 8*π* signal visible in our set-up provides a signature that cannot be confused with these undesired effects.

## Results

### Model Hamiltonian

**Figure 1:**
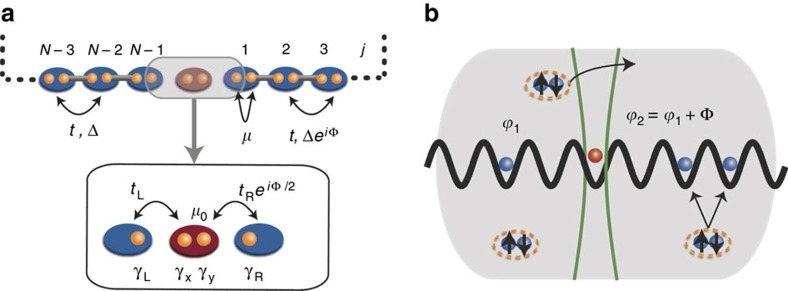
System Hamiltonian and cold atom setting. (**a**) Schematics of the model Hamiltonian equation (1): the central part of the system is magnified in the box at the bottom, where the Majorana degrees of freedom included in the simplified model (equation (2)) are highlighted. (**b**) Implementation in a cold atom system. A 1D optical lattice is coupled to a BEC reservoir that gives rise to the Kitaev Hamiltonian in the chain. An optical barrier acts both to create the impurity site (red) and triggers the Josephson effect in the reservoir itself. The phase difference across the barrier in the reservoir then acts as the phase Φ for the optical lattice.

We consider spinless fermions with field operators 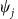, where *j*=0, … *N*−1 labels the sites of a one-dimensional (1D) lattice in ring geometry. The model Hamiltonian reads as


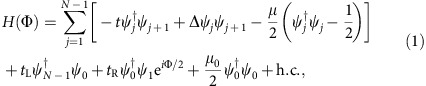


which describes a proximity-induced P-wave superconductor^1^ with pairing Δ, interrupted by an extra site at *j*=0, which is assumed to be not affected by the pairing (Fig. 1a). The hopping strength is denoted by *t* and the chemical potential relative to half-filling by μ. The site at *j*=0 is connected to its neighbours by the hoppings *t*_L_ and *t*_R_, respectively, and has an energy offset *μ*_0_. The phase factor e^*i*Φ/2^ on the hopping between *j*=0 and *j*=1 models a flux that advances the phase of a Cooper pair by Φ when moving around the ring.

For |*μ*|<2*t*, |Δ|>0 and *t*_L_=*t*_R_=0, in the limit of large *N* the system hosts a single pair of zero-energy MQPs^1^, *γ*_L_ and *γ*_R_, which are localized exponentially around *j*=*N*−1 and *j*=1, respectively. All other quasi-particles of the superconductor are gapped, such that 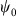 along with *γ*_L_ and *γ*_R_ form a subspace that is energetically detached from the bulk spectrum. To understand the qualitative Φ dependence of equation (1) in the physically relevant regime *t*_L_, *t*_R_<<Δ, *t*, we hence consider a minimal model encompassing the dynamics within this low-energy sector. Decomposing 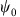 into the Majorana operators 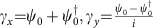, and setting *μ*_0_=0, the effective Hamiltonian then reads as


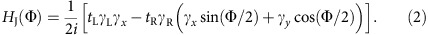


**Figure 2:**
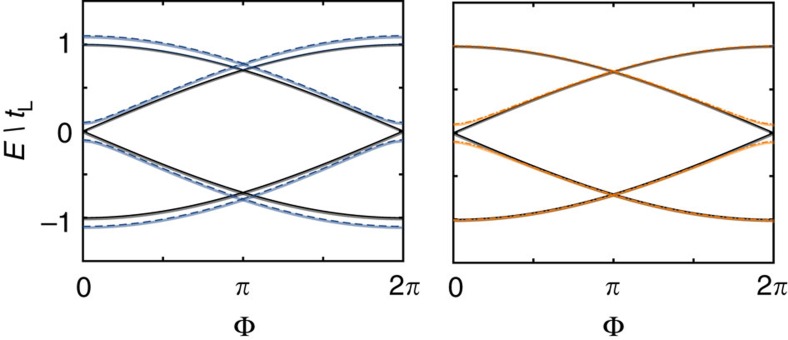
Energy spectrum of the model. The energy spectrum of the minimal model in equation (2) with different parameters, with the width of the lines indicating the deviation from the energy spectrum of the full microscopic model (shifted by an energy constant to lie at the same scale) in equation (1) with (*N*=10, Δ=*t*=10*t*_L_, *μ*=0). Left panel: *t*_R_=*t*_L_, *μ*_0_=0 (black, solid), *t*_R_=1.2*t*_L_, *μ*_0_=0 (blue, dash). Right panel: *t*_R_=*t*_L_, *μ*_0_=0 (black, solid), *t*_R_=*t*_L_, *μ*_0_=0.2*t*_L_ (orange, dot dash). Despite the 4*π* periodicity of the Hamiltonian, adiabatically following the ground state of the spectrum results in an 8*π* periodic pattern. The degeneracies at Φ=*π* are protected by the global 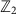 parity symmetry, while the degeneracies at Φ=0 are present for *t*_L_−*t*_R_=*μ*_0_=0.

In Fig. 2, we compare the energy spectra of *H*_J_(Φ) and *H*(Φ). The full qualitative agreement confirms that the effective Hamiltonian *H*_J_(Φ) captures the basic Josephson physics of the full model *H*(Φ). To understand the various level (avoided) crossings in Fig. 2, we first focus on the symmetric case *t*_L_=*t*_R_. At Φ=0, we have 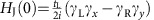, that is, the four Majorana operators form two disjoint pairs giving rise to two single-particle (hole) excitations with energy 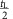 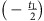. The four possible many-body states then have the energies (−*t*_L_, 0, 0, *t*_L_), which explains the twofold degeneracy at *E*=0. At Φ=*π*, we have 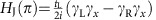, that is, *γ*_L_ and *γ*_R_ are coupled to the same Majorana operator *γ*_*x*_. This gives rise to a zero mode in the single-particle spectrum and the many-body energies are 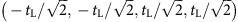 as reflected in the crossings at Φ=*π* in Fig. 2. At Φ=2*π*, we have 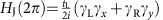, that is, the analogous situation to Φ=0, but with a sign change of a single-particle excitation energy, reflecting the change of the fermion parity in the ground state^1^. At Φ=3*π*, the situation is analogous to Φ=*π* with *γ*_R_→−*γ*_R_. As for Φ=4*π*, we note *H*_J_(4*π*)=*H*_J_(0). However, despite the 4*π* periodicity of *H*_J_, adiabatically following the ground state in Fig. 2 through the various crossings leads to an 8*π* periodic pattern. This is a phenomenon of spectral flow, where the system is pumped to an excited state during one 4*π* cycle of the Hamiltonian, and only returns to the initial state after a second cycle.

We emphasize that the level crossings in Fig. 2 are of quite different physical nature. The crossings between states with different fermion parity at odd multiples of *π* are robust as long as the fermion parity is conserved. By contrast, the crossings at even multiples of *π* require left/right symmetry and a mid-gap state on the additional site: this is realized by tuning the junction parameters, namely, *μ*_0_=0 and symmetric tunnelling *t*_L_=*t*_R_. However, tuning of the bulk parameters within the topological superconductor (TSC) phase supporting the MQPs *γ*_L_ and *γ*_R_ is not required as long as the bulk gap is much larger than *t*_L_ and *t*_R_. In a solid-state setting, the decoherence due to the coupling to phonons implies that observing the non-equilibrium population of the unprotected excited state presents a serious challenge. In contrast, in the cold atom setting proposed here, such decoherence channels are not present, thus stabilizing these effects.

Below we describe how the model given in equation (1) can be realized in systems of AEAs trapped in optical lattices, before discussing in more detail the visibility of the 8*π* Josephson effect in the presence of various imperfections.

### Experimental realization

There are three points required for the realization of our set-up: the implementation of a 1D Kiteav chain, the addition of the single site separating the two ends of the wire, and the time control of the phase Φ. To address these points in a concrete set-up, we consider a system of fermionic AEAs^23^,^24^,^25^,^26^,^27^,^28^,^29^,^30^,^31^,^32^,^33^, trapped in their ^1^*S*_0_ ground state in a 1D lattice. The choice of AEAs allows us to independently trap the ^1^*S*_0_ ground state |*g*〉 and the ^3^*P*_0_ metastable excited state atoms |*e*〉. While our model is for spinless (single species) fermions, the ability to trap two species independently will be, as discussed in more detail below, of crucial use to implement the junction architecture of equation (1). We also note that while AEAs are well known in the experiments for their additional *SU*(*N*) symmetry^34^, here, the choice of AEAs rests on the above reason, and the *SU*(*N*) symmetry plays no role.

We first address the implementation of a 1D Kiteav chain. While the hopping terms (*t*) arise naturally in the lattice, pairing terms (Δ) can be induced by coupling the fermions in the lattice to a BEC reservoir, where a radio-frequency (RF) field is used to break up Cooper pairs directly into neighbouring sites in the lattice, as described in ref. 18.

Second, we address how we can interrupt the chain with a single site. First, at the position *j*=0, a barrier is engineered to inhibit |*g*〉 atoms from being at this site, which splits the Kitaev wire into two. This can be done using a highly focused beam at the so-called anti-magic wavelength, which acts as a sink for |*e*〉, and oppositely on |*g*〉^22^, resulting in the |*e*〉 atoms only being trapped at this site. Thus, the |*e*〉 atom at site *j*=0 acts as the additional site coupling the two ends of the wire. While natural hopping into and out of this site is deterred by this barrier, the tunnelling (*t*_L_ and *t*_R_) are then reintroduced with Raman processes involving a clock transition^35^,^36^,^37^.

Last, we address the time control of the phase Φ. In fact, the barrier which inhibits |*g*〉 atoms to be trapped at *j*=0 also acts as the mechanism that controls the phase Φ. This can be seen as follows. The barrier is turned on via a laser which is highly localized at the *j*=0 position in the optical lattice, but homogenous in the remaining directions and impacts the BEC reservoir, bisecting it into two regions. For a barrier that is only a few times larger than the coherence length of the system, it will act as a thin tunnelling barrier between the two regions. If the two regions have a different Cooper pair density, an ordinary a.c. Josephson effect will occur, giving rise to a relative phase Φ across the junction that oscillates in time^17^. The Josephson frequency *ω*_J_ of this oscillation is proportional to the population imbalance, which constitutes the analogue of a bias voltage in the solid-state context. Due to the proximity effect, this time-dependent phase is inherited by the 1D lattice system, giving rise to the model described in equation (1). Here *ω*_J_ is on the order of the bare trap frequency and can be controlled via the barrier and reservoir parameters.

Within this set-up, there are two main ways to demonstrate the 8*π* periodicity of the Josephson effect by current measurements. First, it is possible to use local interferometric probes, as realized, for example, in ref. 38, or to infer the current behaviour from density measurements^16^,^17^. Second, one can observe clear signatures of the 8*π* periodicity using the relation between the time-dependent momentum distribution and the current operator^39^,^40^. For the model defined in equation (1), the relevant current at the junction is defined by:


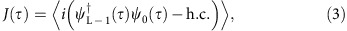


where 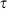 denotes the real time on which the Hamiltonian is dependent via the modulation of the phase 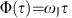, with the Josephson frequency *ω*_J_, such that Φ(0)=0. Since the system we investigate does not display translational invariance, the global current operators cannot be described solely in terms of momentum distribution (momentum is not a good quantum number). Indeed, the total current reads:


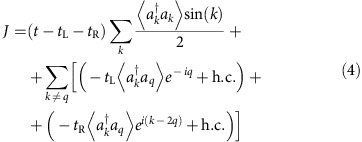


where the presence of the last two terms reflects the fact that momentum is not a conserved quantity. While these terms are not directly accessible in cold atom experiments, it is possible to identify signatures of the 8*π* periodicity via the first term.

**Figure 3:**
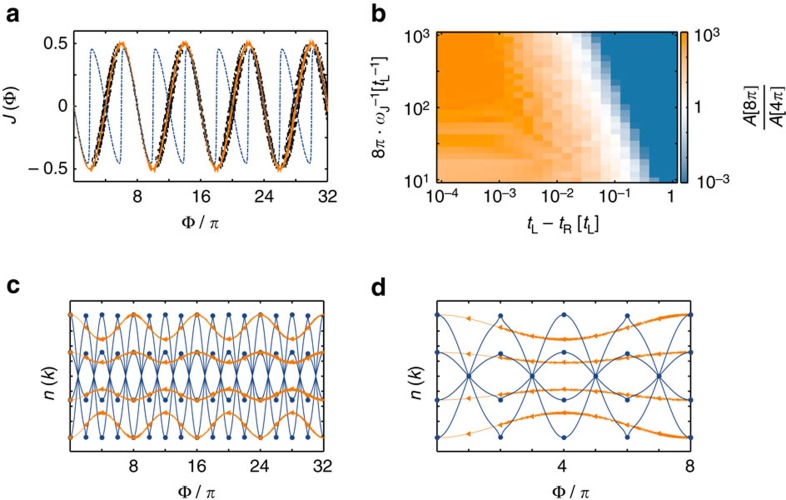
Signatures of 8*π* periodicity. (**a**) Current as a function of Φ for a system with *N*=10, with parameters 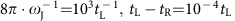 (orange, solid), 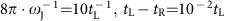 (black, dot dash) and 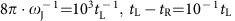 (blue, dash). (**b**) Logarithm of the ratio of the height of the 8*π* 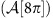 and 4*π* 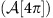 peak of the FFT of the current profile over a range of model parameters. (**c**) Time evolution of the some of the *k*-components of the momentum distribution as a function of Φ for a system with *N*=10, with parameters 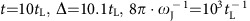 and *t*_R_−*t*_L_=10^−4^*t*_L_ (orange/triangles) and *t*_R_−*t*_L_=10^−1^*t*_L_ (blue/circles). (**d**) Same as in **c** with the time window Φ[0, 8*π*] magnified.

In Fig. 3, we show the time-dependent behaviour of the current (Fig. 3a) and the various components of the momentum distribution (Fig. 3c–d) as a function of time in different parameter regimes, for a system of *N*=10 sites. For system parameters where the current has a dominant 8*π* periodicity (the orange line in Fig. 3a), the momentum components *n*(*k*) individually mirror this. This is shown in the orange (triangle) line of Fig. 3c and d, where the identical parameters have been taken. However, when system parameters are such that the current has a dominant 4*π* periodicity (the blue dashed line in Fig. 3a), the momentum components reflect this. This is shown with the blue (circles) lines of Fig. 3c and d, again with the identical parameters.

We now address the question of the integrity of this 8*π* Josephson effect in our proposed set-up subject to imperfections. First, we address the influence of Hamiltonian imperfections *t*_L_≠*t*_R_ as well as *μ*_0_≠0 leading to avoided crossings in the level spectrum at integer multiples of 2*π* (Fig. 2). We find that Landau–Zener processes restore the 8*π* periodicity of the current at finite bias voltage. Thereafter, we investigate the effect of single-particle losses, induced three-body collisions between particles in the wire and pairs in the reservoir, and dephasing in the framework of a Markovian quantum master equation^41^,^42^.

### Transport dynamics and 8*π* Josephson effect

We study the current through the junction region at site *j*=0, as defined in equation (3). In the limit of perfect adiabatic evolution *ω*_J_→0, at the symmetric parameter point *t*_L_=*t*_R_, *μ*_0_=0, the current will be 8*π* periodic, as indicated in the dispersion relation (Fig. 2); any deviation from this fine-tuned parameter point will cause a gap to open and the adiabatic current will be 4*π* periodic. However, the 8*π* effect is restored at finite *ω*_J_ due to the Landau–Zener effect. This tradeoff between finite *ω*_J_ and finite imperfections is analysed within the coherent time evolution governed by equation (1) in Fig. 3, where we numerically calculate the current *J*(*τ*) as a function of time (Fig. 3a). For small *ω*_J_ and weak imperfections (orange, solid line), the current displays a clear 8*π* periodicity, while increasing imperfections at fixed *ω*_J_ is detrimental (blue, dashed line). However, larger *ω*_J_ allows the system to follow the avoided crossings due to Landau–Zener tunnelling, thus restoring the 8*π* periodicity (black, dot-dashed line).

To provide a quantitative picture of the interplay between imperfections and *ω*_J_, we extract the height of the 8*π* peak (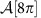) and the 4*π* peak 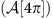 from the Fourier transform of the current over a total phase change of Φ_T_=8*π*. The ratio of these two peaks is shown in Fig. 3b) as a function of *ω*_J_ and (*t*_L_−*t*_R_). At intermediate *ω*_J_, the 8*π* peak dominates over a wide range of parameters: remarkably, even for imperfections of a few per cent, the 8*π* signal is still an order of magnitude stronger than that at 4*π*. This behaviour has been verified with Φ_*T*_=32*π*. Figure 3 shows the data with Φ_*T*_=8*π* to minimize the compound effect of several Landu–Zener crossings (a finite particle loss stabilizes this effect and data at Φ_*T*_=32*π* is shown in these cases, as discussed in the next section).

### Dissipation and open-system dynamics

In addition to imperfections that cause the system to move away from the symmetric point *t*_L_=*t*_R_, *μ*_0_=0, an experimentally relevant imperfection is due to the coupling of the system to its environment. To account for this, we consider two dissipative channels. The first is a single-particle loss at the site *j* with the rate *κ*_*j*_: in cold atom settings, this represents losses due to inelastic collisions with the background BEC reservoir. The second source of dissipation is dephasing due an effective measurement at rate *γ*_*j*_ of the local occupation number 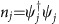 by the environment. This typically represents the effect of spontaneous emission in optical lattice settings. Assuming a weak coupling to a Markovian quantum bath, the time evolution of the system is then governed by the master equation


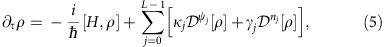


where *ρ* is the density matrix of the system and the superoperator 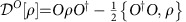 is the Lindblad dissipator for an arbitrary Lindblad jump operator *O*. As long as *γ*_*j*_=0, equation (5) is still quadratic in the field operators 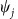 and can be solved numerically efficiently. By contrast, *γ*_*j*_ leads to quartic terms in the master equation (5), which we treat in an exact diagonalization analysis. In what follows, we present results for the full master equation in systems of *N*=10 sites.

To study the impact of a finite *κ*_*j*_≡*κ* on the integrity of the 8*π* effect, we numerically solve the master equation (5) and calculate the current 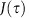 in the presence of finite loss. In such open settings, the system dynamics is now determined by the competition of three energy scales, corresponding to *ω*_J_, the energy scale related to Hamiltonian imperfections and *κ*. At fixed *κ*, one expects a stronger 8*π* signal for intermediate *ω*_J_, since both Landau–Zener tunnelling works at its best even in the presence of imperfections and dissipation becomes detrimental only after many oscillations periods.

**Figure 4:**
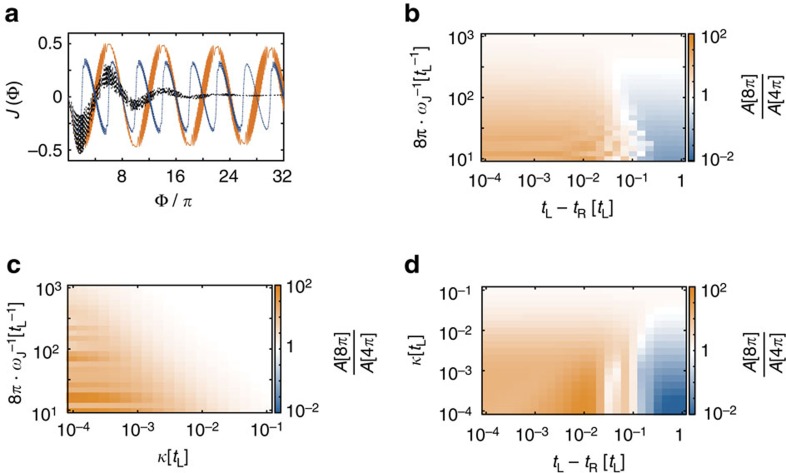
Effect of dissipation on the signatures of 8*π* periodicity. (**a**) Current as a function of Φ for a system with *N*=10, with parameters 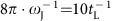 and *κ*=10^−4^*t*_L_, and *t*_L_−*t*_R_=10^−2^*t*_L_ (orange, solid), *κ*=5·10^−3^*t*_L_, *t*_L_−*t*_R_=10^−2^*t*_L_ (black, dot dash), *κ*=10^−4^*t*_L_ and *t*_L_−*t*_R_=*t*_L_ (blue, dash). (**b**–**d**) Ratio of the strength of the 8*π* peak of the FFT of the current profile 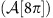 and the 4*π* peak 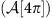 with *κ*=10^−3^*t*_L_ fixed (**b**) *t*_L_−*t*_R_=10^−2^*t*_L_ fixed (**c**) and 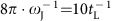 fixed (**d**).

A few examples of the current evolution as a function of time are depicted in Fig. 4a): the main effect of dissipation is to damp the current signal in the system, thus inhibiting transport. However, even for relatively large decay rates (black line, corresponding to decay collision rates of order *κ*≃1 Hz (ref. 18) to be compared with *t*_L_≃200 Hz), the signal stays 8*π* periodic for intermediate timescales (combined with a exponentially decaying envelope).

Following the above analysis, again we quantify the 8*π* effect by extracting the ratio of the 8*π* and the 4*π* peaks from the Fourier spectrum. This ratio is shown for various system parameters and loss rates in Fig. 4b–d, and illustrates the regimes in which the 8*π* signal can be seen. In Fig. 4b), we plot the ratio at fixed *κ*: the best attainable regime is for intermediate values of the velocity, where imperfections are relatively harmless up to values on the order of a few per cent. In Fig. 4c, *t*_L_−*t*_R_ is fixed: here again intermediate speeds work at best, and values of the dissipation of the order of 10^−2^ can be tolerated. Finally, in Fig. 4d, the speed of the ramp is fixed: the signal is solid in the regime of low losses, and, for intermediate values of imperfections, larger values of the losses, *κ*, can be tolerated. The strong signal at these intermediate values of *t*_L_−*t*_R_ is consistent with what is expected from Landau–Zener theory, which predicts an optimal tunnelling rate at intermediate gap values in case of finite dissipation and finite speed.

**Figure 5:**
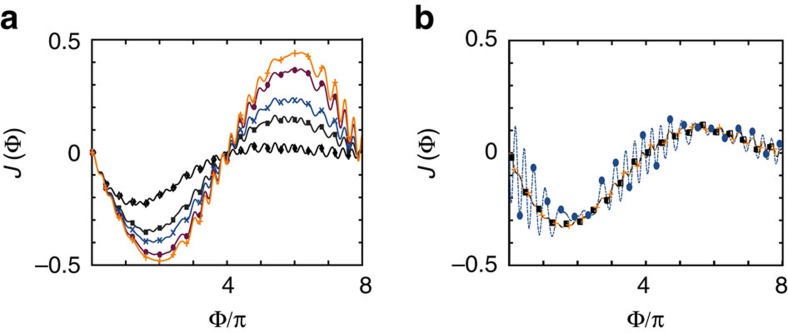
Effect of dephasing and interactions on the current profile. Current as a function of Φ for a system with *N*=10, with parameters 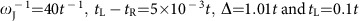. (**a**) Different values of *κ*=*γ*=[0.02, 0.008, 0.005, 0.002, 0.0008] (diamonds, squares, crosses, circles and pluses; ordered top to bottom for short times). (**b**) Fixing *κ*=0.01 and *γ*=0.05, and including the presence of a nearest-neighbour interaction (equation 6), for *U*/*t*_L_=[0.01, 0.1, 1] (orange/crosses/dash, black/squares/solid and blue/circles/dot dash).

We have repeated these calculations in the presence of a finite dephasing rate *γ*. In this case, the system dynamics is not quadratic in the fermions, so our study was limited to system sizes up to *N*=10 sites. A sample of the results is presented in Fig. 5a). Overall, we found that it has qualitatively the same effect as *κ*, which can be understood in terms of the protection of the non-equilibrium excited states. While the decay channel *κ* mixes states with different parity the decay channel *γ* mixes states within the same patrity, both contributing equally through the evolution from 0 to 8*π*. Finally, we have checked how the main effects discussed here are affected by finite-size effects. In the regimes of interest, those effects are negligible at *N*=10. For the *γ*=0 case, we have checked this explicitly for some sample points up to *N*=30, while for the *γ*≠0 case, we have systematically checked consistency with the *N*=8 case.

In summary, the 8*π* periodicity of the current profile is robust to both the Hamiltonian imperfections and the dissipation considered here. Monitoring the evolution for shorter time (for example, for a single 8*π* cycle) can also substantially improve the signal, as in that case the role of particle losses is less detrimental.

### Many-body effects

**Figure 6:**
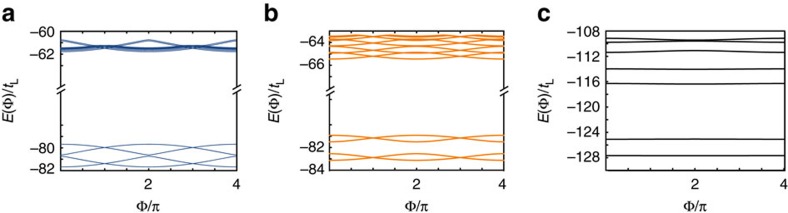
Energy spectrum in the presence of a nearest-neighbour interaction. Parameters Δ=1.01*t*=10*t*_L_, *t*_R_=*t*_L_ and *μ*=*μ*_0_=0 and interaction strength (c.f. equation 6) (**a**) *U*=0.1*t*_L_, (**b**) *U*=*t*_L_ and (**c**) *U*=10*t*_L_. Note in **a** and **b**, the *y* axis has been cut for clarity.

Finally, we consider the effect of a finite interaction on the energy spectrum of the model. At a qualitative level, these interactions have a similar effect as the direct tunnelling between the two superconducting islands: in the presence of interactions, the MQPs are less localized, and the overlap of their wave-functions can lead to a direct interaction between them. To quantitatively study the effect of interactions on our model, we consider a nearest-neighbour interaction of the form


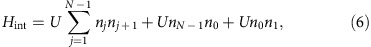


where 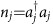. We show the impact of the interaction on both the current pattern (Fig. 5b) and the low-lying spectrum (Fig. 6) for various values of the interaction *U*/*t*. When the interaction strength remains the lowest-energy scale *U*<*t*_L_<*t* (Fig. 6a), a gap will open in the spectrum at even multiples of π, and effects the current measurement similar to the case of other Hamiltonian imperfections *t*_L_≠*t*_R_. As the interaction strength increases, the size of this gap increases, until values of *U*∼*t* the assumption of four low-lying states separated by an energy gap is no longer valid (Fig. 5c). Moreover, we numerically verified that distinct interactions in the bulk and at the junction have similar consequences on the Josephson effect, in agreement with the discussion above (both interactions lead to a delocalization of the MQP wave-functions). Typically, in systems of AEAs for lattice spacings on the order of 250–500 nm the ratio *U*/*t*∼10^−3^ (ref. 32), well within the regime where the 8*π* behaviour can be seen.

## Discussion

The periodicity of the Josephson effect is closely related to the charge of the particles involved in the tunnelling processes. Intuitively, an 8*π* periodicity then corresponds to a fractional charge of 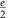, which is the physical picture behind the time-reversal protected fractional Majorana fermions discussed in ref. 43. In contrast, our model does not involve fractional charges, and our effective Hamiltonian *H*_J_(Φ) (equation (1)) is hence 4*π* periodic in Φ, in agreement with the Byers–Yang theorem^44^. The 8*π* Josephson effect in our set-up is a phenomenon of spectral flow: the system is pumped to an excited state after slowly increasing Φ by 4*π*, and returns to the ground state after a second 4*π* cycle. Our work thus shows that an 8*π* periodic signal can also emerge due to non-protected crossings, analogue to what has been shown to occur for the 4*π* effect. However, in the latter case, the accidental 4*π* periodicity occurs when the underlying system is a conventional superconductor; here this 8*π* effect arises when the underlying system hosts ‘normal' 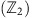 Majorana fermions.

We note that while a 12*π* periodic Josephson effect has been put forward in the context of two connected quantum wires^45^, we emphasize that these effects are dissipationfull, as there is no controllable gap separating the crossing branches of the Josephson junction from the bulk states.

### Data availability

The data that support the findings of this study are available from the corresponding author upon request.

## Additional information

**How to cite this article:** Laflamme, C. *et al*. Non-equilibrium 8*π* Josephson effect in atomic Kitaev wires. *Nat. Commun.* 7:12280 doi: 10.1038/ncomms12280 (2016).
